# 580 Burn Camp in the Midst of the COVID-19 Pandemic

**DOI:** 10.1093/jbcr/irad045.175

**Published:** 2023-05-15

**Authors:** Keisha ONeill, Tiffany Smith, Kathleen Hollowed, Aaron Lesher, Ross Vezin, Ryan Eubanks, Al Nord, Rodney Howell, Steven Kahn

**Affiliations:** South Carolina Burn Center at MUSC, Summerville, South Carolina; South Carolina Burn Center at MUSC, Charleston, South Carolina; South Carolina Burn Center at MUSC, Charleston, South Carolina; South Carolina Burn Center, Charleston, South Carolina; South Carolina Burned Children's Fund, Beaufort, South Carolina; South Carolina Burned Children's Fund, Spartanburg, South Carolina; City of Myrtle Beach Fire Dept, SHALLOTTE, North Carolina; South Carolina Burned Children's Fund, West Columbia, South Carolina; The South Carolina Burn Center/Medical University of South Carolina, Charleston, South Carolina; South Carolina Burn Center at MUSC, Summerville, South Carolina; South Carolina Burn Center at MUSC, Charleston, South Carolina; South Carolina Burn Center at MUSC, Charleston, South Carolina; South Carolina Burn Center, Charleston, South Carolina; South Carolina Burned Children's Fund, Beaufort, South Carolina; South Carolina Burned Children's Fund, Spartanburg, South Carolina; City of Myrtle Beach Fire Dept, SHALLOTTE, North Carolina; South Carolina Burned Children's Fund, West Columbia, South Carolina; The South Carolina Burn Center/Medical University of South Carolina, Charleston, South Carolina; South Carolina Burn Center at MUSC, Summerville, South Carolina; South Carolina Burn Center at MUSC, Charleston, South Carolina; South Carolina Burn Center at MUSC, Charleston, South Carolina; South Carolina Burn Center, Charleston, South Carolina; South Carolina Burned Children's Fund, Beaufort, South Carolina; South Carolina Burned Children's Fund, Spartanburg, South Carolina; City of Myrtle Beach Fire Dept, SHALLOTTE, North Carolina; South Carolina Burned Children's Fund, West Columbia, South Carolina; The South Carolina Burn Center/Medical University of South Carolina, Charleston, South Carolina; South Carolina Burn Center at MUSC, Summerville, South Carolina; South Carolina Burn Center at MUSC, Charleston, South Carolina; South Carolina Burn Center at MUSC, Charleston, South Carolina; South Carolina Burn Center, Charleston, South Carolina; South Carolina Burned Children's Fund, Beaufort, South Carolina; South Carolina Burned Children's Fund, Spartanburg, South Carolina; City of Myrtle Beach Fire Dept, SHALLOTTE, North Carolina; South Carolina Burned Children's Fund, West Columbia, South Carolina; The South Carolina Burn Center/Medical University of South Carolina, Charleston, South Carolina; South Carolina Burn Center at MUSC, Summerville, South Carolina; South Carolina Burn Center at MUSC, Charleston, South Carolina; South Carolina Burn Center at MUSC, Charleston, South Carolina; South Carolina Burn Center, Charleston, South Carolina; South Carolina Burned Children's Fund, Beaufort, South Carolina; South Carolina Burned Children's Fund, Spartanburg, South Carolina; City of Myrtle Beach Fire Dept, SHALLOTTE, North Carolina; South Carolina Burned Children's Fund, West Columbia, South Carolina; The South Carolina Burn Center/Medical University of South Carolina, Charleston, South Carolina; South Carolina Burn Center at MUSC, Summerville, South Carolina; South Carolina Burn Center at MUSC, Charleston, South Carolina; South Carolina Burn Center at MUSC, Charleston, South Carolina; South Carolina Burn Center, Charleston, South Carolina; South Carolina Burned Children's Fund, Beaufort, South Carolina; South Carolina Burned Children's Fund, Spartanburg, South Carolina; City of Myrtle Beach Fire Dept, SHALLOTTE, North Carolina; South Carolina Burned Children's Fund, West Columbia, South Carolina; The South Carolina Burn Center/Medical University of South Carolina, Charleston, South Carolina

## Abstract

**Introduction:**

Previous literature has shown that pediatric burn camps provide numerous benefits to childhood burn survivors. Unfortunately, the COVID-19 pandemic resulted in the inability to host camp for fear of viral transmission to burn survivors and camp personnel alike. A comprehensive burn center, with assistance from the firefighter community, hosted its first burn camp since the onset of the COVID pandemic in August of 2022. A camp staff of 54 volunteers modified the previous camp processes to ensure camp was safe, fun and accessible to 20 pediatric burn survivors.

**Methods:**

This is an academic short report that reviews the process changes necessary to host a safe pediatric burn camp in the midst of the COVID pandemic. All attendees and staff were required to show a negative PCR test result 48 hours prior to arrival at camp. COVID vaccination was encouraged in all participants, however, staff and patients were not required to be vaccinated. Daily symptom tracking was implemented immediately upon arrival to camp. All participants were assigned a thermometer, and daily temperatures and symptom monitoring was performed by the camp director. Masks were mandated when indoors or within close proximity or group activities. There was a quarantine plan in place in the case a camper or staff member became symptomatic, or tested positive for COVID. Lastly, the number of people per cabin decreased from 12 to 8.

**Results:**

In the early planning phase of camp, it was discovered that some of the participants and staff were unable to obtain a PCR test prior to camp. Therefore, onsite testing was implemented to allow testing as the participants and camp staff arrived to camp. The onsite testing facility was available for the entirety of camp in the case anyone became symptomatic. Implementation of additional camp processes resulted in a successful and safe camp. There were no reported cases of COVID during or after camp.

**Conclusions:**

As the Public Health Emergency from COVID-19 continues, group activities, such as summer burn camps, with medically vulnerable populations must be altered to protect all stakeholders. We described the steps taken by one burn-center-run camp that enabled children and firefighter volunteers to participate in the full burn camp experience. Plans for the 2023 burn camp are already in process, and the processes implemented in 2022 will be implemented in all upcoming camps to ensure the children and staff are safe from the transmission of COVID.

**Applicability of Research to Practice:**

N/A

